# The role of hypoxia-inducible factors 1 and 2 in the pathogenesis of diabetic kidney disease

**DOI:** 10.1007/s40620-024-02152-x

**Published:** 2024-12-08

**Authors:** Marcin Kleibert, Kamil Tkacz, Katarzyna Winiarska, Jolanta Małyszko, Agnieszka Cudnoch-Jędrzejewska

**Affiliations:** 1 Department of Experimental and Clinical Physiology, Laboratory of Centre for Preclinical Research, Medical University of Warsaw, 02-097 Warsaw, Poland; 2 Department of Diabetology and Internal Diseases, Medical University of Warsaw, 02-097 Warsaw, Poland; 3 Department of Metabolic Regulation, Institute of Biochemistry, Faculty of Biology, University of Warsaw, Miecznikowa 1, 02-096 Warsaw, Poland; 4 Department of Nephrology, Dialysis and Internal Medicine, Medical University of Warsaw, Banacha 1A, 02-097 Warsaw, Poland

**Keywords:** Diabetic kidney disease, Hypoxia-inducible factor 1, Hypoxia-inducible factor 2, Diabetes mellitus, Pathogenesis

## Abstract

According to the 10th edition of the IDF Diabetes Atlas, 537 million people suffered from diabetes in 2021, and this number will increase by 47% by 2045. It is estimated that even 30–40% of these individuals may develop diabetic kidney disease (DKD) in the course of diabetes. DKD is one of the most important complications of diabetes, both in terms of impact and magnitude. It leads to high morbidity and mortality, which subsequently impacts on quality of life, and it carries a high financial burden. Diabetic kidney disease is considered a complex and heterogeneous entity involving disturbances in vascular, glomerular, podocyte, and tubular function. It would appear that hypoxia-inducible factors (HIF)-1 and HIF-2 may be important players in the pathogenesis of this disease. However, their exact role is still not fully investigated. In this article, we summarize the current knowledge about HIF signaling and its role in DKD. In addition, we focus on the possible effects of nephroprotective drugs on HIF expression and activity in various tissues.

**Graphical abstract:**

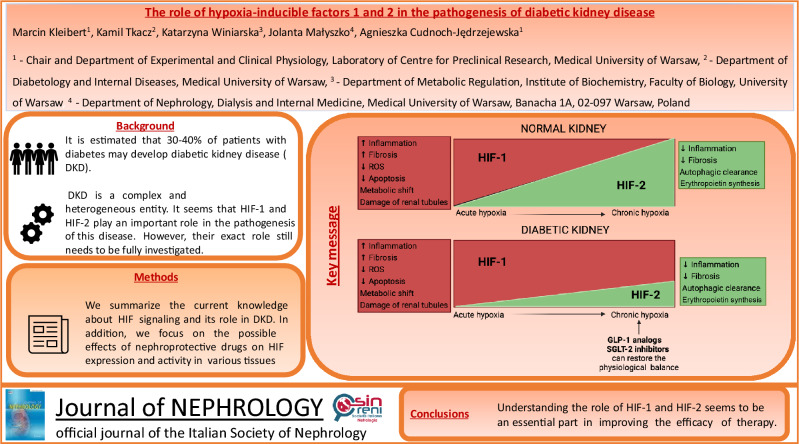

**Supplementary Information:**

The online version contains supplementary material available at 10.1007/s40620-024-02152-x.

## Introduction

**Fig. 1:**
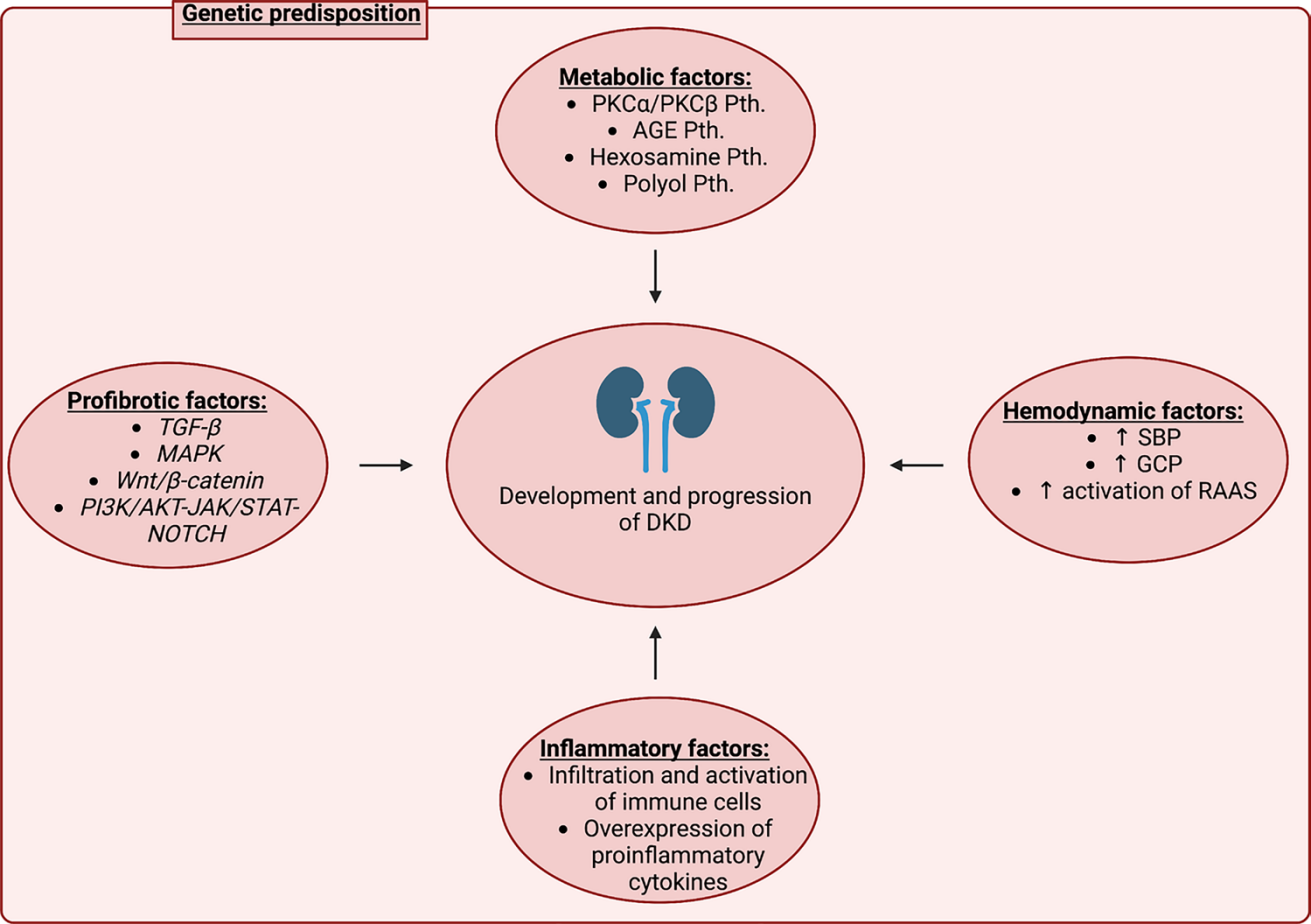
Pathogenesis of diabetic kidney disease (DKD). *AGE* advanced glycation end products, *DKD* diabetic kidney disease, *GCP* glomerular capillary pressure, *JAK*/*STAT* Janus kinase/signal transducers and activators of transcription, *PI3K*/*AKT* phosphatidylinositol-3-kinase/Ak strain transforming, *PKC* protein kinase C, *Pth* pathway, *RAAS* renin–angiotensin–aldosterone system, *SBP* systolic blood pressure, *TGF-β* transforming growth factor beta, *MAPK* mitogen-activated protein kinases. Created with Biorender.com

The precise diagnosis of diabetic kidney disease (DKD) is established in most cases without a kidney biopsy in diabetic patients who experience albuminuria and kidney function impairment. The pathogenesis of DKD is not completely understood. Diabetic kidney disease is considered a complex and heterogeneous entity with various overlapping etiologic pathways, involving cellular responses to hyperglycemia, and hypoxia, in addition to the responses in vascular, glomerular, podocyte, and tubular function [1] (Fig. 1).

High glucose levels lead to advanced glycation end-products and reactive oxygen species synthesis. Moreover, both advanced glycation end-products and reactive oxygen species result in the activation of intercellular signaling for profibrotic and proinflammatory gene expression leading to the synthesis of various cellular injury mediators, such as hypoxia-inducible factor (HIF)-1 [2]. Besides the role of hyperglycemia in the development of micro-and macroangiopathic complications, it should be emphasized that hyperinsulinemia, insulin resistance, and lipotoxicity may also be associated with pathogenic mechanisms and possibly contribute to the histopathology variation between diabetes type 1 and type 2 [3]. In addition, the diabetic milieu is responsible for renin–angiotensin–aldosterone system activation as well as a variety of downstream mediators, resulting in kidney hypertrophy, enhanced renal plasma flow, and filtration fraction, which together lead to an abnormally increased glomerular filtration rate (GFR) [4]. It is often referred to as “glomerular hyperfiltration” as both “whole kidney GFR” and “single nephron GFR” are elevated in the early stages of diabetes [5]. Furthermore, the activation of these mediators may lead to an impaired response to hypoxia and an increased amount of HIF-1. It should also be delineated that besides diabetic glomerular hyperfiltration, including primary vascular events, tubular growth, hyperreabsorption, and tubuloglomerular communication also play a role as part of a “tubulocentric” concept of early diabetic kidney disease. This “tubulocentric” concept of DKD identifies the unique early growth phenotype of the proximal tubule as a potential target for the prevention of early tubular hyperreabsorption and glomerular hyperfiltration [6]. As shown previously, the mercapturate pathway, mainly expressed in renal proximal tubular cells, that is responsible for metabolic detoxification, might represent a target for early intervention in DKD [7]. More importantly, it links tubulointerstitial inflammation, fibrosis, oxidative stress, hypoxia, and kidney dysfunction. In addition, as stressed by Zeni et al., inhibition of proximal tubule glucose transport via sodium-glucose cotransporter 2 (SGLT-2) is nephroprotective in diabetic patients [8]. Moreover, several tubular biomarkers were considered direct indicators of proximal tubule injury, including N-acetyl-β-D-glucosaminidase, Neutrophil Gelatinase-Associated Lipocalin, and Kidney Injury Molecule-1, In addition, other functional proximal tubule biomarkers, such as Urine free Retinol-Binding Protein 4 and Cystatin C, reflect impaired reabsorption of filtered proteins.

Moreover, it seems that an imbalance between the amount of HIF-1 and HIF-2 may be one of the mechanisms accelerating the progression of DKD. In this article, we summarize the current knowledge about the role of HIF signaling in DKD, which can be an underestimated player in this disease.

### Hypoxia-inducible factors

HIF-1 and HIF-2, together with the lesser known HIF-3, belong to the family of hypoxia-inducible factors. The members of this family (HIFs) are heterodimers composed of a regulatory subunit α and a constitutively expressed subunit β/ARNT. The latter subunit is the same for all HIFs, but there are three different isoforms of the regulatory subunit α. While the structures of HIF-1α and HIF-2α appear to have a lot in common, HIF-3α homology is the lowest within the family [9].

Each of the four subunits mentioned above possesses a domain enabling DNA binding (basic helix-loop-helix; bHLH) and domains responsible for heterodimerization *i.e.* Per-ARNT-Sim homology (PAS-A and PAS-B) domains. However, only HIF-1α and HIF-2α exhibit the presence of two characteristic transactivation domains (C-TAD and N-TAD) separated by an inhibitory domain [10].

The domain which is crucial for the canonical regulation of HIFs, *i.e.* oxygen-dependent degradation domain, is characteristic of all the regulatory subunits: HIF-1α, HIF-2α, and HIF-3α, but does not occur in HIF-β [10]. The most important feature of the oxygen-dependent degradation domain is the presence of one of the prolyl residues that may be hydroxylated, as described below. Nuclear localization of HIFs is determined by the presence of nuclear localization signal (C-NLS and N-NLS) domains [10].

**Fig. 2:**
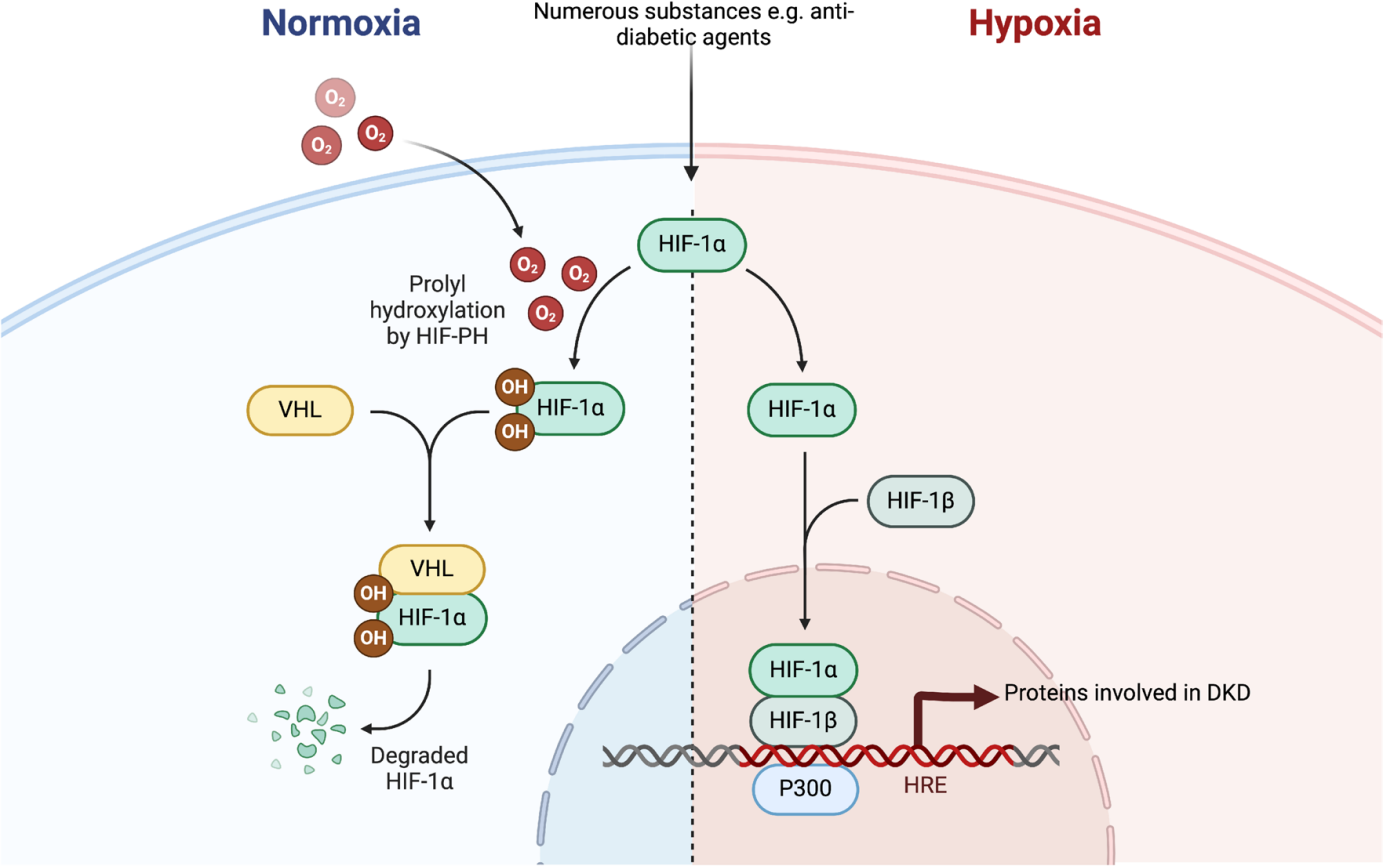
Hypoxia-inducible factor 1 signaling in normoxia and hypoxia. *DKD* diabetic kidney disease, *HIF-1α* hypoxia-inducible factor 1 subunit alfa, *HIF-1β* hypoxia-inducible factor 1 subunit beta, *HIF-PHs* hypoxia-inducible factor prolyl hydroxylases, *HRE* hypoxia responsible element, p300 transcription factor-coactivator, *VHL* von Hippel-Lindau protein. Created with Biorender.com

As depicted in Fig. 2, under normoxic conditions, either HIF-1α (prolyl residues 402 and 564) or HIF-2α (prolyl residues 405 and 531) are hydroxylated by prolyl hydroxylases 1–3. The activity of these enzymes depends directly on the supply of oxygen, one of their substrates (2-oxoglutarate presence is also necessary). Then, the von Hippel-Lindau protein (VHL) binds to the hydroxylated subunit, leading to the recruitment of the other proteins of the ubiquitin ligase E3 complex. Finally, HIF-1α ubiquitination directs it to degradation in a proteasome [11]. In hypoxia, as a result of the absence of the crucial substrate, prolyl hydroxylases remain inactive, the regulatory subunit α (either 1α or 2α) avoids degradation and interacts with HIF-β, creating functional heterodimeric HIF which then binds to its coactivator p300/CBP. Finally, the whole complex binds to specific DNA sequences called hypoxia-responsible element (5′-RCGTG-3′, where R stands for adenine or guanine) and activates transcription of the upstream genes [9].

Moreover, in normoxia, another hydroxylase (in this case asparaginyl) –factor inhibiting hypoxia-inducible factor 1 (HIF-1) is also activated. It is responsible for the hydroxylation of asparaginyl residue 803 in HIF-1α and of asparaginyl residue 847 in HIF-2α, disabling HIF interactions with p300/CBP and – consequently – additionally lowering the hypoxia-responsible element-containing gene transcription [12].

### HIF-1 and HIF-2 involvement in DKD pathogenesis

Hypoxia was observed in the kidneys of animal models with induced diabetes [13] as well as in the blood oxygen level-dependent (BOLD)-MRI study of patients with DKD [14]. Several mechanisms may be responsible for renal hypoxia accompanying diabetes. Arteriolar dysfunction, which is frequently present in diabetes, may result in structural changes impairing blood flow and causing local ischemia in the kidney [15]. Oxidative stress, exacerbated by diabetes, may lead to increased oxygen consumption in the kidneys [16]. Another significant cause of excessive oxygen consumption is elevated sodium reabsorption in proximal tubules coupled with glucose reabsorption by SGLT-2. In addition to increased sodium reabsorption and oxygen consumption, transport efficiency is decreased and oxygen usage subsequently increases [17].

**Fig. 3:**
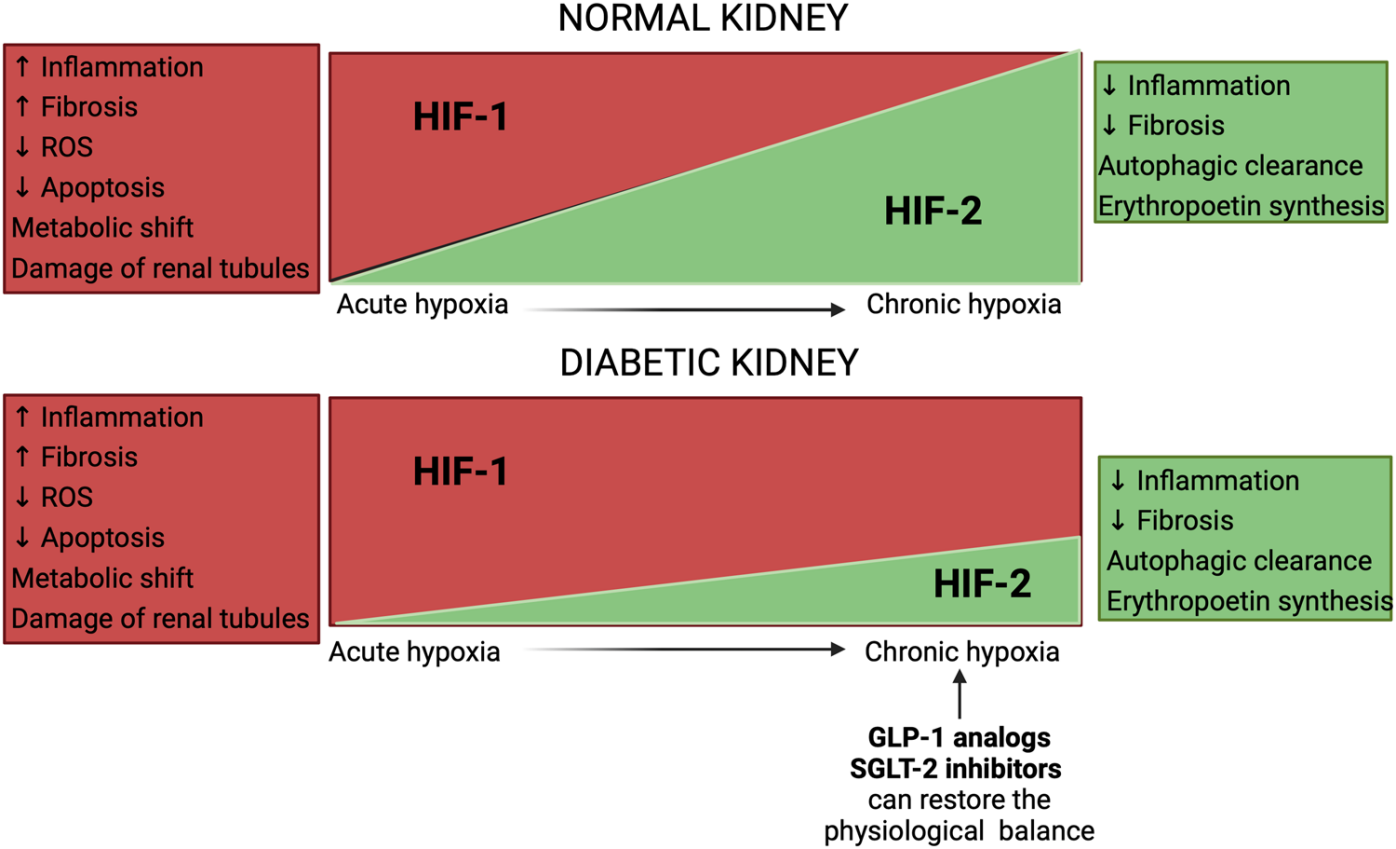
Differences in HIF 1 and 2 balance between normal and diabetic kidneys. *GLP-1* glucagon-like peptide 1, *HIF* hypoxia-inducible factor, *ROS* reactive oxygen species, *SGLT-2* sodium/glucose transport protein 2. Created with Biorender.com

Acute hypoxia induces HIF upregulation, whose role is to mitigate hypoxia-related injury [18]. HIF-1 has a protective role in acute hypoxia, inhibiting reactive oxygen species accumulation and mitochondrial apoptosis in renal tubular epithelial cells [19]. Both HIF-1 and HIF-2 participate in the response to hypoxia, but HIF-2 activity begins later than HIF-1 and it continues to be active after 48–72 h of hypoxia [20]. This switch from HIF-1 to HIF-2 expression constitutes an adaptive mechanism to prolonged hypoxia (Fig. 3) [21]. However, in DKD, chronic hypoxia may lead to dysregulation of those mechanisms. HIF-1α expression differs in various renal cell types. Its activity in diabetes is elevated in glomerular mesangial cells and inhibited in proximal tubular cells [22]. Overall, HIF-1α activity is higher in the diabetic kidney than in the healthy kidney. However, the activity is submaximal to the level of hypoxia that is observed in DKD, since HIF-1α expression is, to some extent, impaired by hyperglycemia. Consequently, hypoxia-induced mechanisms, i.e. vascular endothelial growth factor (VEGF) response through the HIF pathway, are dysregulated [23]. Jiang et al. showed that HIF-1α proximal tubule-specific knockout in a diabetic mouse model caused increased mitochondrial fragmentation, reactive oxygen species production, and apoptosis of renal cells. These mechanisms were reversed by HIF-1α overexpression or by the agonists of heme oxygenase-1, the product of the HIF-1 target gene [24]. HIF-1α deficient mice with induced diabetes also displayed decreased body weight, increased fasting blood glucose, urinary albumin, and systolic blood pressure compared to a diabetic mouse model with HIF-1α expression [25]. HIF-1 takes part in the regulation of sodium transport by controlling the expression of epithelial sodium channels and may decrease the epithelial sodium channel abundance in the condition of hypoxia. Subsequently, it may preserve oxygenation and reduce reactive oxygen species production [26].

In addition, HIF isoform ratios may have an impact on diabetic kidney disease development. Oxidative stress, chronic renal hypoxia, and impaired nutrient deprivation signaling result in a local imbalance between HIF-1α and HIF-2α expression. HIF-1 activity may be markedly increased compared to HIF-2 [27]. This sustained upregulation of HIF-1 has detrimental effects on renal tissue. Its role in improving vascularization by angiogenesis is associated with the development of inflammation and profibrotic mechanisms [28]. It was reported that the kidneys of diabetic mice were more susceptible to renal fibrosis caused by unilateral ureteral obstruction due to significantly higher HIF-1α levels compared to nondiabetic kidneys [29]. Diabetic kidneys were also characterized by a significantly higher apoptosis rate and interstitial macrophage infiltration [29]. Other mechanisms may also contribute to increased fibrosis in diabetic kidneys. HIF-1α induces ferroptosis, which is an iron-dependent form of cell death. It may aggravate DKD, and was shown to take part in the damage of renal tubules in animal diabetic models [30]. HIF-1α accumulation contributes to the metabolic shift and induces aberrant glycolysis, which may enhance the fibrotic and inflammatory effect [31]. The upregulation of HIF-1α in the models of chronic kidney disease also resulted in NOTCH-1 activation which is known for its profibrotic effect in the kidney [32]. In vitro and in vivo studies demonstrated that hypoxia-induced HIF-1 activation leads to osteochondrogenic differentiation of vascular smooth muscle cells, which causes vascular calcification [33]. PDK4 upregulation by HIF-1α is one of the key mechanisms of vascular calcification [34]. Arteriolar dysfunction and vascular calcification in DKD may aggravate hypoxia, and subsequently increase HIF-1 activation. Moreover, advanced glycation end-products accelerate vascular calcification partly through the HIF-1 pathway [35]. HIF-1 activation by daprodustat, which is a prolyl hydroxylase inhibitor, increased vascular calcification measured in the aorta of CKD mice [36]. In the recent study by Xu et al. in a rat model of CKD, the positive staining area of HIF-1α, a marker for hypoxia was significantly larger than that in the sham group, and losartan alleviated the expression of HIF-1α in CKD rats [37].

In contrast, HIF-2 plays the opposite role in the progression of the inflammatory state. It exerts anti-inflammatory and anti-fibrotic effects, promotes autophagic clearance, and induces erythropoietin (EPO) production in the diabetic kidney, especially when the activation begins at the late stage of chronic kidney disease [38]. Thus, HIF-1α overexpression in mesangial cells of diabetic kidneys, compared to the limited expression of HIF-2α, aids the progression of kidney disease. Decreased activity of HIF-2α and, consequently, inhibition of the synthesis pathway may explain why CKD is often associated with anemia [39]. HIF-2α plays a protective role in vascular disorders, which often lead to diabetic complications. It was found that upregulation of HIF-2α inhibits the development of atherosclerotic plaques and neointimal proliferation [40]. Moreover, activation of HIF-2α in the endothelium of renal arteries protects from hypoxia-induced renal damage in murine models of hypoxic kidney injury [41]. The exact mechanisms of HIF-2-induced renoprotection are not known and require more research.

Little is known about HIF activity in patients with DKD. In the published literature, elevated serum HIF-1α concentration showed a significant correlation with the progression of nephropathy in Saudi patients with type 2 diabetes mellitus (T2DM) [42]. On the other hand, Zapora-Kurel et al. found that levels of HIF-1α are negligible in patients with type 2 DM and were related to age only in patients with estimated GFR (eGFR) values ≥ 60 mL/min/1.73 m^2^ [43].

### Antidiabetic drugs and HIF activity regulation

Large placebo-controlled studies, which included patients with DKD or non-diabetic patients with CKD, proved the efficacy of SGLT-2 inhibitors [44] and glucagon-like peptide 1 (GLP-1) analogs [45] in decreasing the risk of kidney failure and/or cardiovascular death. The impact of these drugs on HIF activity is discussed in detail below. Table 1 summarizes the most important mechanisms of HIF-1 and HIF-2 regulation by antidiabetic drugs and their renoprotective effects. Among a wide range of other drugs adopted in diabetes therapy, only DDP-4 (dipeptidyl peptidase 4) inhibitors have been reported to affect HIFs.

**Table 1:** Mechanisms of HIF-1 and HIF-2 regulation by antidiabetic drugs and their effects on renoprotection

Drug		↓ HIF-1	↑ HIF-2
SGLT-2 inhibitors	Hypothetic mechanism	Direct inhibition of HIF-1α [50, 56] Inhibition of HIF-1 downstream genes (heme oxygenase 1) [50, 56] Upregulation of complement receptor type 1-related protein y (Crry) [51]	Suppression of hepcidin and ferritin and iron mobilization [47] Decrease in oxidative stress [47] Increased SIRT1 signaling [47]
	Effect	Decreased inflammation and fibrosis (decreased interstitial fibronectin) [50, 53] Limited tubular injury [53] Suppression of aberrant glycolysis [54] Suppression of ferroptosis [56]	Increased EPO production [47] Decreased inflammation and fibrosis [47]
GLP-1 analogs	Hypothetic mechanism	Decrease in HIF-1α mRNA and protein (exact mechanism not known) [62]	(–)
	Effect	Decreased inflammation and fibrosis (also by TGF-β and Egr-1) [62]	(–)
DDP-4 inhibitors	Hypothetic mechanism	(–)	PHD3 inhibition [49]
	Effect	(–)	Decreased mesangial expansion, tubular dilation, hydropic degeneration and basement membrane thickness [49] Decreased KIM-1 levels [49]

(–) No data describing the exact mechanism and effect

*Crry* complement receptor type 1-related protein y,* DPP-4* dipeptidyl peptidase-4,* EPO* erythropoietin,* Egr-1* early growth response protein-1,* GLP-1* glucagon-like peptide-1,* HIF-1* hypoxia-inducible factor-1,* HIF-2* hypoxiainducible factor-2,* KIM-1* kidney injury marker-1,* PHD3* prolyl hydroxylase domain 3,* SGLT-2* sodium–glucose cotransporter-2,* SIRT1* sirtuin 1,* TGF-β* transforming growth factor β

### SGLT-2 inhibitors

They are the most commonly investigated drugs in HIF-dependent renoprotection. Various studies have described their role in EPO synthesis. The analysis of proteomics from patients involved in the EMPEROR study demonstrated significantly increased EPO levels after 12 weeks of empagliflozin intervention [46]. Packer proposed four hypotheses explaining the role of SGLT-2 inhibitors in increased EPO production, and two of them involve HIF-2α pathways [47]. SGLT-2 inhibitors may increase iron mobilization by suppressing proinflammatory hepcidin and ferritin, thus promoting HIF-2α signaling. Another hypothesis says that SGLT-2 inhibitors enhance nutrient deprivation signals through the SIRT1 pathway which acts to stimulate HIF-2α. SGLT-2 inhibitors also decrease excessive oxygen consumption by inhibiting the sodium-glucose transport in renal proximal tubules. Subsequently, the oxidative stress decreases. Moreover, SGLT-2 inhibitors may ameliorate the metabolic changes in DKD and fibrosis progression. These hypotheses might indicate how SGLT-2 inhibitors increase HIF-2α activity and its renoprotective actions. However, there are no studies describing direct stimulation of HIF-2α by SGLT-2 inhibitors in the kidney.

The majority of in vitro and in vivo studies demonstrate the suppressive role of SGLT-2 inhibitors on HIF-1 and its downstream gene expression, as well as its stimulating role on HIF-2α [27]. Inada et al. investigated the expression of HIF-1α and HIF-2α in various cells of a diabetic kidney mouse model. The authors observed increased expression of both isoforms in glomerular and tubular cells of diabetic kidneys. Canagliflozin lowered the overexpression of HIF-1α after 33 weeks of  administration and nullified the expression after 50 weeks. After 33 weeks of the intervention, HIF-2α was expressed to some extent in the nuclei of glomerular cells, but it disappeared after 50 weeks of treatment [48]. This is inconsistent with other studies and reviews which described the beneficial effect of SGLT-2 inhibitors by increasing the concentration and activity of HIF2-α [49].

In one study, dapagliflozin was compared to losartan (an ACE inhibitor) in a diabetic mouse model. Both drugs equally reduced albuminuria, glomerular tuft area, mesangial expansion, and mesangial matrix hyperplasia. However, only dapagliflozin was shown to improve tubulointerstitial fibrosis and immune infiltration, and to almost nullify the HIF-1α level in proximal renal tubules [50]. In addition, dapagliflozin attenuates complement over-activation in diabetic mice by upregulating the complement receptor type 1-related protein y. It is associated with the suppression of HIF-1α. This effect is blocked by the addition of dimethyloxallyl glycine which stabilizes the expression of HIF-1α and causes its accumulation in mouse proximal tubule epithelial cells [51]. Dapagliflozin alone or combined with losartan was found to be equally effective upon assessment of hypoxia-related profibrotic factors CTGF and PDGF expression [52]. Another study demonstrated that the SGLT-2 inhibitor, luseogliflozin, attenuated HIF-1α expression in proximal cortical tubular cells, which was also associated with a decreased interstitial fibronectin level and limited tubular injury [53]. Empagliflozin administration to streptozotocin-induced diabetic mice reverted fibrotic changes in their kidneys and suppressed aberrant glycolysis associated with HIF-1α accumulation [54]. A similar effect was achieved even in contrast-induced acute kidney injury, where dapagliflozin suppressed the HIF-1 pathway, having a beneficial influence on the affected kidneys [55]. Interestingly, the authors of the previous study observed that the administration of the prolyl hydroxylase inhibitor molidustat, which is an HIF-1α stabilizer that increases its concentration, resulted in a metabolic shift similar to cells treated with high concentrations of glucose. Moreover, molidustat reversed the beneficial effect of dapagliflozin on metabolic changes [50]. On the other hand, studies have also shown that the addition of HIF-1 stabilizers may mitigate metabolic changes in the diabetic kidney [55]. Below we describe the role of prolyl hydroxylase inhibitors in DKD.

Another mechanism of the renoprotective role of SGLT-2 inhibitors includes amelioration of ferroptosis, which is an iron-dependent type of cell death. It was demonstrated that dapagliflozin suppresses ferroptosis-related changes via inhibiting HIF-1α and heme oxygenase 1, the downstream gene of HIF-1α [56].

Most authors state that SGLT-2 inhibitors limit oxygen consumption in the kidney and cause HIF-1 downregulation. In contrast, one in vitro study showed the opposite effect of empagliflozin on HIF-1 regulation. The authors demonstrated the increased level of HIF-1α upon empagliflozin administration to HK-2 cells (human epithelial cells of renal proximal tubules) cultured under conditions of high glucose concentration [57] and suggested that it may play a crucial role in the renoprotective action of SGLT-2 inhibitors, i.e., in their antifibrotic and antiproliferative effect. This is inconsistent with other studies and was not confirmed in in vivo studies.

### GLP-1 analogs

It was suggested that HIF-1 activates glycolytic pathways in β-cells after chronic stimulation of GLP-1 receptors, which may be a significant mechanism in glucose-dependent insulin secretion [58]. Linagliptin was shown to have an anti-angiogenic effect mediated by the HIF-1α/VEGF pathway in diabetic retinopathy. In addition, this effect was independent of GLP-1 receptors [59]. Under conditions of obesity, HIF-1 may also regulate liver degradation of the GLP-1 hormone by inducing DPP-4 expression. It was reported that HIF-1α knockout caused an increase in GLP-1 concentration and improved glucose tolerance in mice [60]. On the other hand, HIF-2 participates in lipid-mediated GLP-1 secretion by inducing the lipid sensor–membrane receptor GPR-40 in the small intestine. As a consequence, GLP-1 secretion is increased, which improves glucose tolerance [61].

The renoprotective mechanisms of GLP-1 analogs in DKD are not known. However, the HIF-1 signaling pathway may also play a role in this process. Preclinical studies showed that liraglutide administration was associated with a significant decrease in HIF-1α mRNA and HIF-1α protein levels in the renal tubules and glomeruli of mice with DKD. The protective effect was also related to the regulation of transforming growth factor β (TGF-β) and early growth response-1 (Egr-1) expression, which are proteins associated with inflammation and fibrosis [62].

### DDP-4 inhibitors

Recently, a dipeptidyl peptidase-IV inhibitor (vildagliptin) was compared to an SGLT-2 inhibitor (empagliflozin) in diabetic rats. The authors measured the effects of renal HIF-1α and HIF-2α mRNA content and prolyl hydroxylase 1 and prolyl hydroxylase 2 levels. Only empagliflozin was found to significantly lower HIF-1α expression, but both drugs significantly increased HIF-2α expression in diabetic kidneys. In addition, the treatment caused prolyl hydroxylase 3 inhibition, which is the key regulator of HIF-2. Vildagliptin and empagliflozin reversed pathological changes such as mesangial expansion, tubular dilation, hydropic degeneration, and decreased basement membrane thickness. HIF-1α and HIF-2α gene expression demonstrated a strong positive and negative correlation, respectively, with these structural changes and urinary levels of kidney injury marker 1 protein (KIM-1). It was consistent with the renoprotective role of both drugs. Nonetheless, empagliflozin proved to have better effects than vildagliptin and was associated with a stronger influence on HIF isoform levels [49]. Interestingly, after ischemia/reperfusion injury, saxagliptin, another DDP-4 inhibitor, was shown to enhance angiogenesis by stimulating the HIF-1 pathway [64]. Therefore, regulation of HIF isoforms may depend on the duration of renal injury and other factors.

## Pharmacological regulation of HIF signaling and its role in DKD

Regulation of the HIF pathway is considered a therapeutic target of DKD therapy. In this section, we have summarized the most important information about available HIF regulators.

### Cobalt chloride

It is one of the classic hypoxia mimetics that activates both HIF-1 and HIF-2 in animal models of CKD [65]. The compound was shown to normalize renal oxygen consumption in the DKD model [66]. Moreover, cobalt chloride directly binds to and activates HIF-2 [67]. The effects mediated by HIF-2 may be potentiated by the simultaneous action of cobalt chloride activating SIRT1, which deacteylates HIF-2, increasing its activity, and promoting the expression of HIF-2 target genes, including erythropoietin. Consequently, cobalt chloride treatment ameliorates proinflammatory and profibrotic pathways and prevents injury in diabetic kidneys [68]. Cobalt chloride has not been studied in patients with DKD.

Besides cobalt chloride, the 5-(1-acetyl-5-phenylpyrazolidin-3-ylidene)-1,3-dimethylbarbituric acid (PyrzA) was recently reported to increase HIF intracellular levels. This substance stabilizes HIF-1α by decreasing its prolyl residue hydroxylation. Unfortunately, it has not been studied in the context of DKD [69].

### HIF-prolyl hydroxylase inhibitors

This group of compounds is the only one (among those described in this section) that is approved for clinical use (the first approval for roxadustat was in China in 2018). It is worth mentioning that none of the available HIF-prolyl hydroxylase inhibitors is HIF subtype-specific, so their administration not only stimulates EPO production but also angiogenesis, switch from aerobic to anaerobic metabolism, activation of M1 macrophages, expression of proinflammatory cytokines, etc. [27]. However, it would appear that most of the metabolic effects related to the administration of these drugs are caused by the stabilization of HIF-2α. Indeed, this group of drugs may play an important role in the modification of the course of DKD.

The impact of HIF-prolyl hydroxylase inhibitors has been investigated in many pre-clinical and clinical studies. In studies on mouse models, it was proved that the first approved HIF-prolyl hydroxylase inhibitor, roxadustat (FG-4592), protects against ischemia/reperfusion-induced acute kidney injury by inhibiting the mitochondrial damage pathway [70]. In another study, Riopel et al. showed that the administration of pan-HIF-prolyl hydroxylase inhibitor (JNJ-42905343) can improve glycemic control by increasing insulin and decreasing glucagon sensitivity in animal models of diabetes, regardless of body weight change. This is caused by an HIF-2-induced increase of *Irs2* and cyclic AMP-specific phosphodiesterase gene expression [71]. In an animal model of metformin-associated lactic acidosis in CKD, it was shown that the administration of roxadustat or REC2923 was associated with a decrease in the expression of the proinflammatory cytokine, interleukin 6 (IL-6). It may have an organ-protective effect and contribute to better survival [72]. Xie et al. showed that the addition of roxadustat into the cell culture of rat glomerular endothelial cells is related to a significant reduction of apoptotic cells after exposure to hyperglycemia. Additionally, the authors showed that this may be caused by the stabilization of HIF-1α and HIF-2α [73]. Wang et al. demonstrated that regulating HIF-2/HIF-1 activity by roxadustat improves vascular calcification in CKD rats [74]. The restoration of HIF-1 function can reduce reactive oxygen species production despite persistent hyperglycemia. It may be considered an intervention that protects against apoptosis and renal injury in diabetes [75]. Roxadustat protects against ischemia/reperfusion-induced acute kidney injury by inhibiting the mitochondrial damage pathway in mice [76]. A randomized controlled trial (RCT) showed that roxadustat could raise hemoglobin levels and decrease the rate of red blood cell transfusions among non-dialysis-dependent and dialysis-dependent patients [77, 78]. Moreover, roxadustat is effective in both CKD patients with and without diabetes. However, the dose required to achieve the target hemoglobin level (110-130 g/L) may be higher in diabetic patients than in non-diabetics [79].

It was shown that HIF-prolyl hydroxylase inhibitor administration reduces fibrosis and damage in the tubular interstitium by ameliorating changes induced by chronic hypoxia. The inhibition of HIF degradation also ameliorated tubular cell apoptosis in an animal model of CKD (Thy-1 nephritis and 5/6 nephrectomy). Additionally, Sugahara et al. used the *ob/ob* mouse model to assess the impact of enarodustat administration on DKD. They noted that the treated group was characterized by lower body weight, reduced blood glucose levels with improved insulin sensitivity, lower total cholesterol levels, higher adiponectin levels, and less adipose tissue, as well as a tendency for lower macrophage infiltration. Moreover, enarodustat administration to *ob/ob* mice also decreased albuminuria and ameliorated glomerular epithelial and endothelial damage. The authors demonstrated that these effects of enarodustat administration were directly mediated by suppression of C–C motif chemokine ligand 2/monocyte chemoattractant protein-1 (CCL2/MCP-1) production via HIF-1 activation in mesangial cells [80].

There is also evidence that some HIF-pyruvate dehydrogenase inhibitors may protect from high-fat, diet-induced obesity and dyslipidemia. These effects could be partly attributed to reduced inflammation in adipose tissue, which was associated with decreased TNF-α expression. Moreover, the authors noted higher glomerular macrophage infiltration and mesangial expansion (the type IV collagen-positive area was smaller in treated animals). However, the administration of enarodustat did not have an impact on the structural derangement of foot processes of podocytes and plasma creatinine levels in animal models [81]. On the other hand, mice with systemic HIF activation by the other pharmacological prolyl hydroxylase inhibitor, FG-4497, and Phd2 hypomorphic mice showed reductions in white adipose weight, adipocyte area, and macrophage infiltration [82].

Interestingly, the positive impact of HIF-prolyl hydroxylase inhibitors was not observed in all clinical trials. In one of the RCTs, which was designed to assess the efficacy of enarodustat, no significant changes in eGFR, or urine protein were observed [83]. However, the efficacy in achieving target hemoglobin levels and safety was comparable to darbepoetin-alfa [84]. There are no clinical studies dedicated to DKD with enarodustat. In another phase 2a clinical trial (with vadadustat), the authors did not observe changes in serum levels of VEGF, cholesterol, cystatin C, or triglycerides as was shown in pre-clinical studies [85]. However, we still do not have long-term observations which assess the impact of HIF-prolyl hydroxylase inhibitor administration on cancer development, which can be an important factor limiting their clinical use. Daprodustat, molidustat, and vadadustat can also be considered alternatives to darbepoetin-alfa in non dialysis-dependent and dialysis-dependent patients [86]. Additionally, daprodustat was evaluated as a topical therapy for diabetic foot ulcers. However, it failed to confirm clinical significance due to the small study group in the phase 1 clinical trial [87]. A summary of the clinical application and efficacy of all registered HIF-prolyl hydroxylase inhibitors can be found in the recently published experts’ statement [86].

## Conclusions

The growing number of patients with DKD is becoming an increasing challenge for the healthcare systems. Understanding the role of HIF-1 and HIF-2 seems to be essential for improving the efficacy of therapy. Increased expression of HIF-1 target genes, with a concomitant decrease in HIF-2 activity, is typical of patients with DKD. Many of the newly registered drugs positively affect the balance between the activity of these two proteins, which contributes to improving the prognosis of patients. These positive effects are expressed, among other things, by an increase in erythropoietin synthesis, and a reduction of oxidative stress. However, there is still a lack of clinical studies investigating the direct role of SGLT-2 inhibitors, and GLP-1A in HIF-1 and HIF-2 signaling. In addition, the efficacy of HIF-prolyl hydroxylase inhibitor therapy in patients with DKD is still not fully understood, necessitating clinical trials targeted to this selected group of patients.

## Supplementary Information

Below is the link to the electronic supplementary material.

Supplementary file1 (DOCX 15 KB)

## Data Availability

Not applicable.
