# Supplementary material for: The role of hypoxia-inducible factors 1 and 2 in the pathogenesis of diabetic kidney disease
Source: J Nephrol. 2024 Dec 8;38(1):37–47. doi: 10.1007/s40620-024-02152-x (PMC11903585; doi:10.1007/s40620-024-02152-x)
Supplement: Supplementary file 1 — Supplementary file1 (DOCX 15 KB) [file 40620_2024_2152_MOESM1_ESM.docx]

**Supplementary Information, Additional references**

71. Riopel, M., et al., *Inhibition of prolyl hydroxylases increases hepatic insulin and decreases glucagon sensitivity by an HIF-2α-dependent mechanism.* Molecular Metabolism, 2020. **41**.

72. Oyaizu-Toramaru, T., et al., *Targeting Oxygen-Sensing Prolyl Hydroxylase for Metformin-Associated Lactic Acidosis Treatment.* Molecular and Cellular Biology, 2023. **37**(16).

73. Xie, R.-y., et al., *Salidroside and FG-4592 ameliorate high glucose-induced glomerular endothelial cells injury via HIF upregulation.* Biomedicine & Pharmacotherapy, 2019. **118**.

74. Wang, Y., et al., *Roxadustat ameliorates vascular calcification in CKD rats by regulating HIF‐2α/HIF‐1α.* Environmental Toxicology, 2023. **39**(4): p. 2363-2373.

75. Xu, C., et al., *Repression of hypoxia-inducible factor-1 contributes to increased mitochondrial reactive oxygen species production in diabetes.* eLife, 2022. **11**.

76. Zhang, M., et al., *Roxadustat (FG-4592) protects against ischaemia/reperfusion-induced acute kidney injury through inhibiting the mitochondrial damage pathway in mice.* Clin Exp Pharmacol Physiol, 2022. **49**(2): p. 311-318.

77. Fishbane, S., et al., *Roxadustat for Treating Anemia in Patients with CKD Not on Dialysis: Results from a Randomized Phase 3 Study.* Journal of the American Society of Nephrology, 2021. **32**(3): p. 737-755.

78. Akizawa, T., et al., *Phase 3, Randomized, Double-Blind, Active-Comparator (Darbepoetin Alfa) Study of Oral Roxadustat in CKD Patients with Anemia on Hemodialysis in Japan.* Journal of the American Society of Nephrology, 2020. **31**(7): p. 1628-1639.

79. Ito, H., et al., *Relationship Between the Effect of Roxadustat and Comorbid Diabetes in Non-dialyzed Chronic Kidney Disease Patients: A Retrospective Observational Study.* Cureus, 2023.

80. Sugahara, M., et al., *Prolyl Hydroxylase Domain Inhibitor Protects against Metabolic Disorders and Associated Kidney Disease in Obese Type 2 Diabetic Mice.* Journal of the American Society of Nephrology, 2020. **31**(3): p. 560-577.

81. Saito, H., et al., *Inhibition of prolyl hydroxylase domain (PHD) by JTZ-951 reduces obesity-related diseases in the liver, white adipose tissue, and kidney in mice with a high-fat diet.* Laboratory Investigation, 2019. **99**(8): p. 1217-1232.

82. Rahtu-Korpela, L., et al., *HIF prolyl 4-hydroxylase-2 inhibition improves glucose and lipid metabolism and protects against obesity and metabolic dysfunction.* Diabetes, 2014. **63**(10): p. 3324-33.

83. Akizawa, T., et al., *A Placebo-Controlled, Randomized Trial of Enarodustat in Patients with Chronic Kidney Disease Followed by Long-Term Trial.* American Journal of Nephrology, 2019. **49**(2): p. 165-174.

84. Akizawa, T., et al., *A Phase 3 Study of Enarodustat in Anemic Patients with CKD not Requiring Dialysis: The SYMPHONY ND Study.* Kidney International Reports, 2021. **6**(7): p. 1840-1849.

85. Martin, E.R., et al., *Clinical Trial of Vadadustat in Patients with Anemia Secondary to Stage 3 or 4 Chronic Kidney Disease.* American Journal of Nephrology, 2017. **45**(5): p. 380-388.

86. Ku, E., et al., *Novel anemia therapies in chronic kidney disease: conclusions from a Kidney Disease: Improving Global Outcomes (KDIGO) Controversies Conference.* Kidney International, 2023. **104**(4): p. 655-680.

87. Olson, E., et al., *Randomized Phase I Trial to Evaluate the Safety, Tolerability, Pharmacokinetics, and Pharmacodynamics of Topical Daprodustat in Healthy Volunteers and in Patients With Diabetic Foot Ulcers.* Clinical Pharmacology in Drug Development, 2019. **8**(6): p. 765-778.
